# The association between flagellin producers in the gut microbiota and HDL-C level in humans

**DOI:** 10.3389/frmbi.2023.1287369

**Published:** 2023-11-09

**Authors:** Jensen H. C. Yiu, Jieling Cai, Samson W. M. Cheung, Karie Tsz-Ching Chin, Chi Fai Chan, Edward S.C. Ma, Rakesh Sharma, Bernhard Dorweiler, Connie W. Woo

**Affiliations:** ^1^ State Key Laboratory of Pharmaceutical Biotechnology, Li Ka Shing Faculty of Medicine, The University of Hong Kong, Hong Kong, Hong Kong SAR, China; ^2^ Department of Pharmacology and Pharmacy, Li Ka Shing Faculty of Medicine, The University of Hong Kong, Hong Kong, Hong Kong SAR, China; ^3^ Proteomics and Metabolomics Core, Centre for PanorOmic Sciences, Li Ka Shing Faculty of Medicine, The University of Hong Kong, Hong Kong, Hong Kong SAR, China; ^4^ Department of Vascular and Endovascular Surgery, University of Cologne, Faculty of Medicine and University Hospital Cologne, Cologne, Germany; ^5^ Micon Analytics, Toronto, ON, Canada

**Keywords:** flagellin, HDL-C, TLR5, flagellated microbiota, Eubacterium rectale, Roseburia intestinalis, Roseburia inulinivorans

## Abstract

The gut microbiota can be beneficial and harmful to cardiovascular health depending on the mechanisms. The interaction between gut microbiota-derived flagellin and toll-like receptor 5 in hepatocytes, resulting in apolipoprotein A1 (ApoA1) production, brings forth a cardiovascular benefit to the host. Here, the association between flagellated microbiota and high-density lipoprotein-cholesterol (HDL-C) in humans was explored. Through sex-based gut microbiota analysis of two population-based cohorts, the 500 Functional Genomics Project (500FG) and Chinese cohorts, we found positive correlations between the capacity to produce flagellins in the gut microbiota and HDL-C in females of the 500FG and males of Chinese cohorts. *Eubacterium rectale, Lachnospira pectinoschiza*, *Roseburia intestinalis* and *Roseburia inulinivorans* were crucial species for such correlations. Diverse types of flagellins and TLR5, but not NAIP/NLRC4, flagellin-engaging receptors, were detectable by proteomic analysis of the human liver. However, not all flagellated bacteria yield the same degree of such benefit because of differences in the penetration of flagellins where other factors such as geographics and diets may play important roles.

## Introduction

Increasing evidence has shown an association between the gut microbiota and cardiovascular risks (Wang et al., 2011; Koeth et al., 2013; Li et al., 2016). A population-based cohort study suggested that the gut microbiome is correlated with body mass index (BMI), triglyceride and high-density lipoprotein-cholesterol (HDL-C) levels (Fu et al., 2015). HDL-C levels were found to be positively correlated with species richness in a study focusing on the connections between habitual diet, gut microbiomes, and cardiometabolic markers (Asnicar et al., 2021). However, these studies have yet to address what properties of the gut microbiota contribute to such correlations. Men and women have distinctive lipid profiles and patterns of the gut microbiota (Ding and Schloss, 2014; Sinha et al., 2019); therefore, we speculate that the separate analysis of the gut microbiota in men and women may give us hints on whether and how the gut microbiota affects HDL-C levels.

Our previous study using a mouse model showed that a high-fat diet increased the abundance of flagellated bacteria in the gut, and the flagellins released by the gut microbiota activated toll-like receptor 5 (TLR5), a flagellin-engaging receptor, resulting in the elevation of ApoA1 and HDL-C levels in mouse and human hepatocytes (Yiu et al., 2020). ApoA1, a key component on HDL, facilitates reverse cholesterol transport by which cholesterol is transported from peripheral cells to the liver, and regression of atherosclerotic lesions is one of the beneficial outcomes (Ouimet et al., 2019). The present study aimed to investigate whether there are a positive correlation between flagellated microbiota and HDL-C levels and an accumulation of flagellins in the liver in humans. We separately analyzed the gut microbiota of females and males from the 500 Functional Genomics (500FG) Project, a population-based cohort, and discovered a positive correlation between the flagellum-producing capacity in the gut microbiota and HDL-C level in female participants. *Roseburia intestinalis* and *Roseburia inulinivorans* were the two major species contributing to this correlation. In contrast, in a Chinese cohort in which males had relatively higher HDL-C levels, the positive correlation between flagellum-producing capacity and HDL-C level was stronger in males than females, which was mainly attributed to *Eubacterium rectale* and *R. intestinalis*. On the other hand, both flagellins derived from flagellated microbiota and TLR5 protein were detectable in the liver. By comparing the sex differences in these two cohorts, we found that flagellated microbiota partially explained HDL-C levels, but these bacteria did not equally lead to such benefits, likely due to variations in flagellin infiltration caused by other factors.

## Results

### Sex differences in the correlations between the gut microbiota and lipoproteins of the 500FG cohort

**Figure 1:**
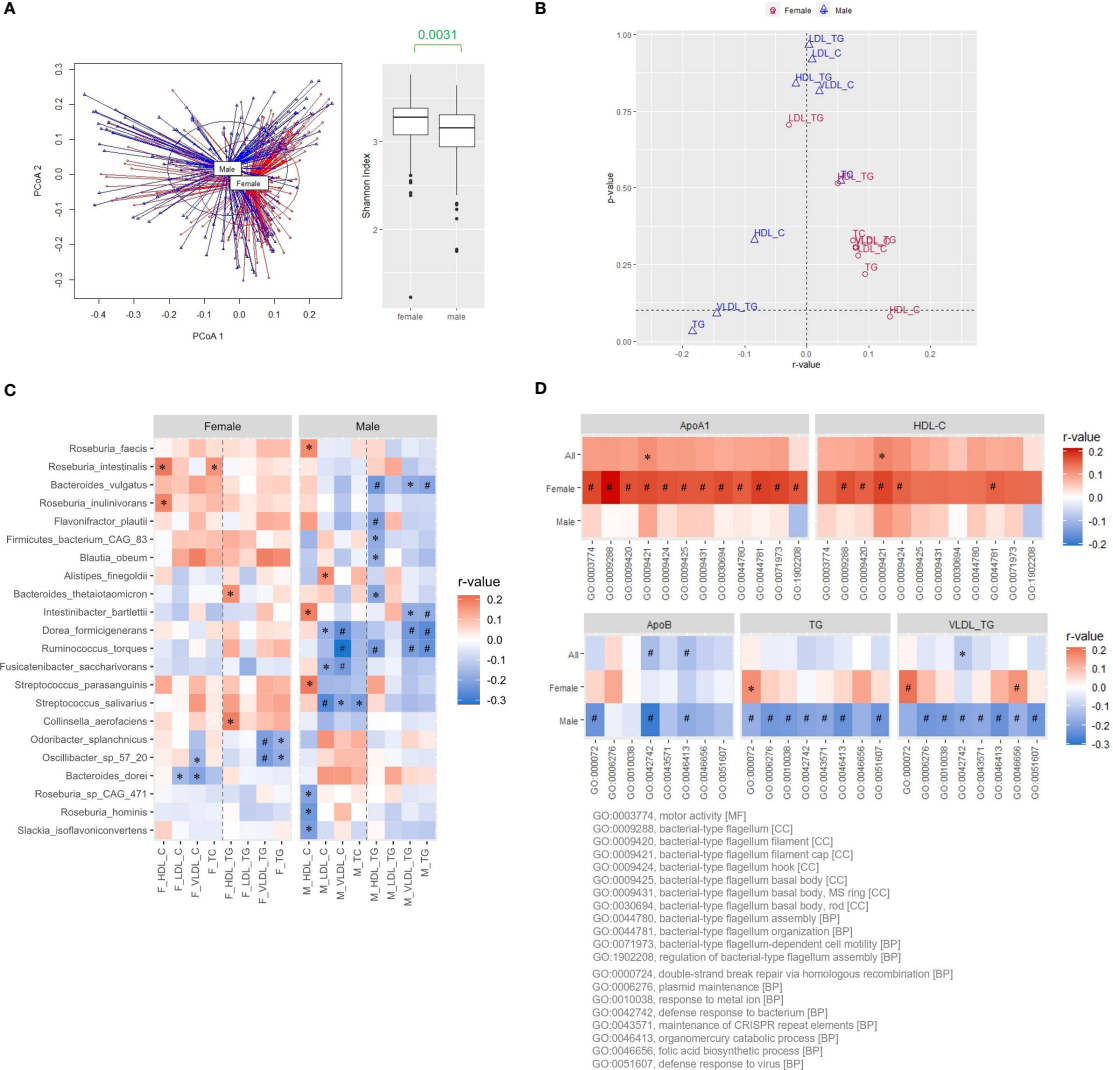
Positive association of microbial diversity with ApoA1 and HDL-C levels in females of the 500FG cohort. **(A)** β and α diversities evaluated by principal coordinate analysis (PCoA) and Shannon index, respectively, of the gut microbiota from female and male subjects of the 500FG cohort. Mann-Whitney U test. **(B)** Correlation of α diversity with the lipid contents of various lipoproteins. **(C)** Correlation between the abundance of bacteria and lipid content of various lipoproteins. Species with statistically significant positive correlations with HDL-C levels in females, and those with statistically significant negative correlations with TG levels in males were shown. **(D)** Correlations of flagellum-related pathways with ApoA1 and HDL-C levels and correlations of survival-related pathways with ApoB, TG, and VLDL-TG levels. Spearman correlation was adjusted for age and BMI for all, and the use of oral contraceptives for females followed by the Benjamini-Hochberg procedure for multiple comparisons in **(B, C)**. ^*^ *P*<0.05, ^#^ *P*<0.05 and *q*<0.2.

After controlling for data quality and completeness, 347 samples (193 females and 154 males) of the 500FG cohort with shotgun metagenomic sequences of the gut microbiome and comprehensive lipid profiles were analyzed (Schirmer et al., 2016). Significantly higher HDL-C, HDL-triglycerides (TG), low-density lipoprotein (LDL)-TG, and total cholesterol (TC) levels were observed in females, while higher age, BMI, very low-density lipoprotein (VLDL)-C, and VLDL-TG levels were observed in males (Mann-Whitney U test, *P*<0.05, **Supplementary Table 1**). Distinctive microbial patterns (permutational multivariate ANOVA (PERMANOVA), *P*=0.001) and intra-sample diversity (α-diversity evaluated using Shannon diversity index) were detected between the females and males (**Figure 1A**). We further analyzed the correlations between α-diversity and various plasma lipid parameters in both sexes separately with adjustment for BMI and age, as well as oral contraceptive intake for females because oral contraceptives are known to affect lipoproteins (Godsland et al., 1990). In females, α-diversity was positively correlated with most of the plasma lipoproteins, among which HDL-C level showed the strongest association (**Figure 1B**). In contrast, negative correlations between VLDL-TG and TG levels were observed in males (**Figure 1B**). Among the 67 bacteria present in ≥50% of the 500FG cohort, the abundance of 41 species showed positive correlations with the HDL-C level in females, and *R. intestinalis* and *R. inulinivorans* were the species with the strongest correlation. Conversely, 49 out of the 67 species had negative correlations with TG levels in males, and *Ruminococcus torques*, *Dorea formicigenerans* and *Intestinibacter bartlettii* showed the strongest correlation (**Figure 1C**, **Supplementary Table 2**). As expected, since TG in circulation is mainly carried by VLDL particles, the pattern of the correlations with VLDL-TG levels was similar to that with TG levels in males (**Figure 1C**).

### Association between flagellum-related pathways and HDL-C level in the females of 500FG cohort

Next, the correlations between lipoproteins and pathways from Gene Ontology (GO) functional enrichment analysis of the gut microbiota were examined (Ashburner et al., 2000; Aleksander et al., 2023). We ranked the GO terms positively correlated with HDL-C levels by the magnitude of the correlation coefficients for females. Among the top 100 GO terms, 36 were categorized as biological processes, 11 as cellular components, and 53 as molecular functions. Four of the 36 biological-process terms and 7 of the 11 cellular-component terms represented flagellum-related (FLA) pathways (**Supplementary Figure 1A**, **Supplementary Table 3A**). The same analysis was performed for males by ranking GO terms negatively associated with TG levels. In contrast, the top 100 terms were mainly under molecular function ontology and were mostly related to bacterial growth and adaptation to habitats (**Supplementary 1A**, **Supplementary Table 3B**). The correlations between FLA pathways and plasma ApoA1 levels, the functional constituent of HDL particles, were further evaluated, and the correlations were even stronger than those with HDL-C levels in females, particularly for the GO:0009288 term bacterial-type flagellum (**Figure 1D**, **Supplementary Figure 1B**, **Supplementary Table 4**). One of the concerns of the gut microbiota from the perspective of cardiovascular risks is the potential induction of systemic inflammation. Hence, we also evaluated the correlation between FLA pathways and various cytokines. These pathways consistently showed positive correlations with the levels of adiponectin, an adipokine known for its anti-inflammatory effect, in both males and females (**Supplementary Figure 2**). The level of interleukin-18 binding protein (IL18BP), a decoy receptor of proinflammatory IL18, showed weak positive correlations in females but negative correlations in males (**Supplementary Figure 2**). In contrast, proinflammatory cytokines only had weak or no correlation with FLA pathways, regardless of sex (**Supplementary Figure 2**). These data suggest a positive correlation between flagellated microbiota and HDL-C levels in the female participants of the 500FG cohort.

### Detection of flagellins derived from fecal flagellated microbiota in human liver

**Figure 2:**
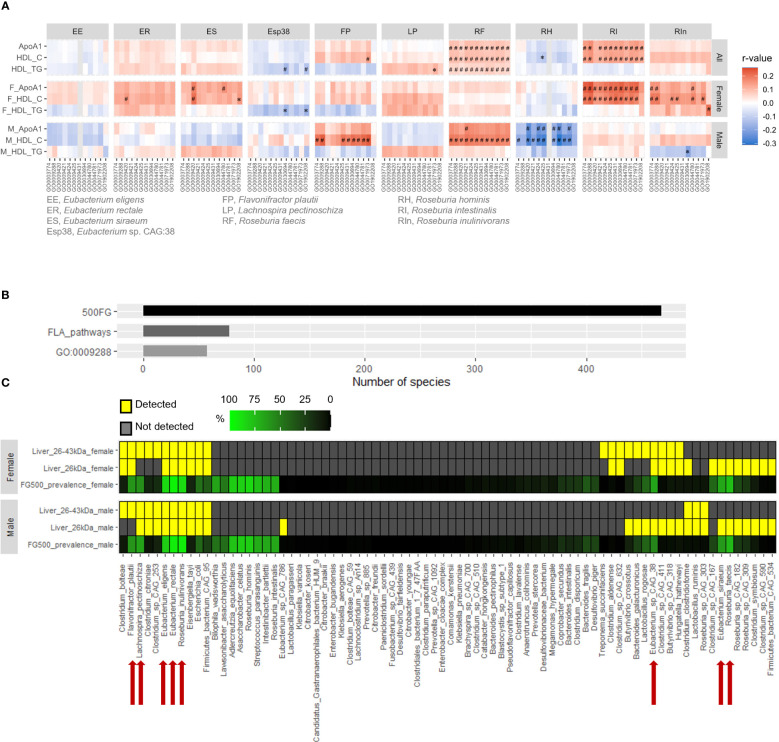
Correlation of flagellated species with HDL-C levels and detection of their flagellins in human livers. **(A) **Correlations of the abundance of flagellum-related (FLA) pathways from individual flagellated bacteria with ApoA1, HDL-C and HDL-TG levels in males and females of the 500FG cohort. Spearman correlation adjusted with age and BMI for all, and the use of oral contraceptives for females followed by Benjamini-Hochberg procedure for multiple comparison. ^*^ *P*<0.05, ^#^ *P*<0.05 and *q*<0.2. **(B)** The numbers of bacterial species detected in the 500FG cohort, contributed to FLA pathways and to the GO:0009288 term. **(C)** The prevalence of bacterial species that contributed to FLA pathway in the 500FG cohort and their presence in human liver tissues (*n*=5 donors per sex). Red arrows indicate the bacteria FLA pathways of which were positively correlated with HDL-C level in **(A)**.

As flagellins, the building blocks of bacterial flagella, vary among different species of bacteria, we wanted to determine which bacterial species contributed to the positive correlation between FLA pathways and ApoA1 and HDL-C levels. The FLA pathways derived from *R. intestinalis* and *R. inulinivorans* showed the strongest correlations with ApoA1 and HDL-C levels in females, followed by *E. rectale* and *E. siraeum*, whereas those from *Flavonifractor plautii* and *R. faecis* were more strongly correlated in males (**Figure 2A**, **Supplementary Table 5**). Conversely, the abundance of FLA pathways from *E. siraeum* and *Lachnospira pectinoschiza* was positively correlated with HDL-TG levels in males (**Figure 2A**, **Supplementary Table 5**). We previously demonstrated that flagellins can be detected in the mouse liver (Yiu et al., 2020). Using two different anti-flagellin antibodies raised against flagellins from bacteria of distinct phyla, we detected bands between 26 kDa and 43 kDa in human liver samples (**Supplementary Figure 3**). In addition to the enterohepatic gateway, the content in the gastrointestinal tract can enter the lymphatic system which is the drainage of visceral lymph nodes to adipose depots. However, compared to the liver, the flagellin level in retroperitoneal fat was negligible (**Supplementary Figure 3**), suggesting the accumulation of flagellins in the human liver. Next, two sets of liver protein samples, one with sizes around 26 kDa and the other with sizes between 26 and 43 kDa, were subjected to proteomic analysis using a flagellin proteome database created based on those contributing to the FLA pathways in the 500FG cohort (Tyanova et al., 2016). In the gut microbiota analysis, 468 bacterial species were detected in the 500FG cohort, 78 of which contributed to the FLA pathways, with 58 species contributing to the readout of the GO:0009288 term (**Figure 2B**). In contrast, the hepatic flagellins at 26 kDa were derived from 23 species in females and 27 species in males, whereas those from 26 to 43 kDa were derived from 21 species in females and 14 species in males (**Figure 2C**, **Supplementary Table 6**). This suggests that only a small portion of flagellated bacteria have flagellins that can penetrate the gastrointestinal tract, and most of the infiltrated flagellins are smaller in size. Moreover, only eight of these species were present in at least 50% of individuals in the 500FG cohort, including *E. rectale*, *E. eligens*, *E. siraeum*, *E.* sp. CAG:38, *L. pectinoschiza*, *F. plautii*, *R. faecis* and *R. inulinivorans* (**Figure 2C**; **Supplementary Table 6**). However, the flagellins derived from *R. intestinalis*, whose flagellin-producing capacity showed the highest correlation among female participants in the 500FG cohort, were not detectable in the liver samples of either sex (**Figure 2C**, **Supplementary Table 6**). Taken together, these findings suggest that for most of the flagellated bacteria that showed positive correlations with ApoA1 and HDL-C levels in the 500FG cohort, their flagellins were detectable in the liver.

### Different abundance of flagellated species in the gut microbiota of different ethnicities

**Figure 3:**
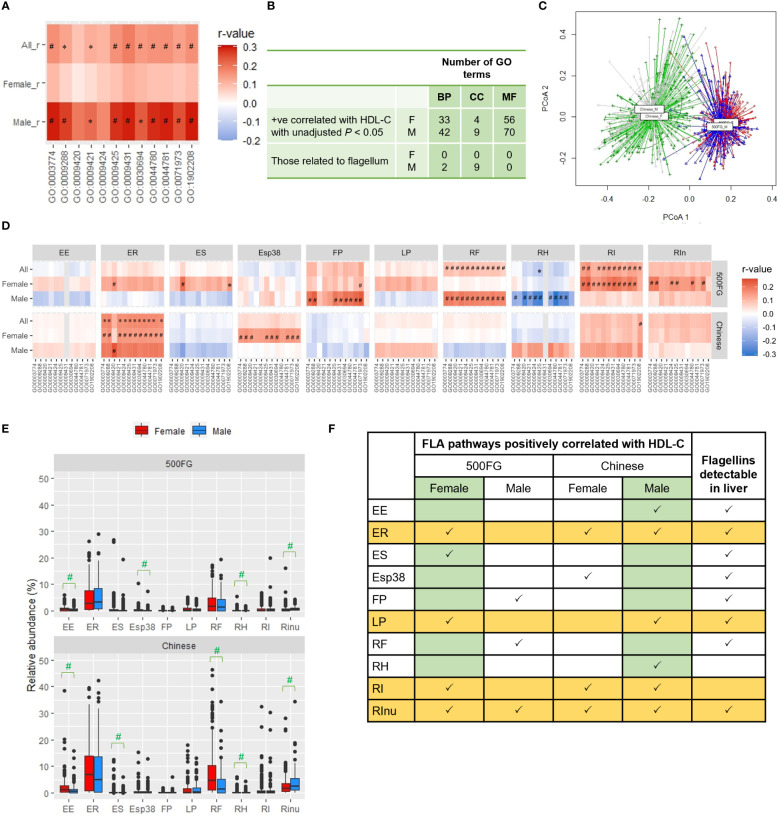
Comparison of correlations between the 500FG and Chinese cohorts. **(A)** Correlation of HDL-C level with the flagellum-related (FLA) pathways in the Chinese cohort. **(B)** The number of gene ontology (GO) terms positively correlated with HDL-C level in the Chinese cohort with unadjusted *P*<0.05 in the 3 sub-ontologies: biological process (BP), cellular component (CC) or molecular function (MF). **(C)** β diversity evaluated by principal coordinate analysis (PCoA) of gut microbiota from female and male subjects of the 500FG and Chinese cohorts. **(D)** Correlations of the abundance of FLA pathways from individual flagellated bacteria with HDL-C level in males and females of the 500FG and Chinese cohorts. (The data of 500FG is derived from **Figure 2A**). **(E)** Relative abundances of different flagellated bacteria in males and females from the 500FG cohort and the Chinese cohort. **(F)** Summary of the correlations between FLA pathways and HDL-C level for individual flagellated species and the detection of their flagellins in the liver. The full names of the FLA pathways and bacteria are listed in **Figures 2**, **3**, respectively. Spearman correlation adjusted with age and BMI in **(A, D)**. Mann-Whitney U test in **(E)**. *P* values were adjusted with Benjamini-Hochberg procedure for multiple comparison. ^*^ *P*<0.05, ^#^ *P*<0.05 and *q*<0.2. .

We previously observed a positive correlation between the abundance of genes representing the flagellin-based flagellum in feces and HDL-C level in overweight males with HDL-C levels ≥ 1 mM, but not <1 mM, in a small Chinese cohort (Yiu et al., 2020). In the 500FG cohort, more than 66% of the males had HDL-C levels <1 mM; hence, the absence of correlation was consistent with our previous data (Yiu et al., 2020). Nonetheless, when we examined another cohort, a Chinese cohort in which 85% of males in the urbanized population had an HDL-C level ≥ 1 mM (**Supplementary Table 7**) (Sun et al., 2021), higher positive correlations between FLA pathways and HDL-C levels were observed in males than in females (**Figure 3A**). Among all the GO terms mapped in these Chinese males, 120 were positively correlated with HDL-C level with an unadjusted *P*<0.05, including 42 biological process terms, 9 cellular-component terms, and 70 molecular-function terms (**Figure 3B**, **Supplementary Table 8**). Similar to the findings from the females in the 500FG cohort, the cellular component terms were all flagellum-related in these Chinese males (**Figure 3B**, **Supplementary Table 8**). The gut microbiota of this Chinese cohort displayed a distinctive pattern that differed significantly from that of the 500FG cohort, and sex differences were also observed (PERMANOVA, *P*=0.001) (**Figure 3C**). The FLA pathways derived from *R. intestinalis* and *E. rectale* which had stronger correlations with HDL-C levels in females of the 500FG cohort, showed stronger correlations in these male participants, but positive correlations between the FLA pathways from *F. plautii* and *R. faecis* were no longer observed in these males (**Figure 3D**). This Chinese cohort showed various distinctive patterns: higher abundances of *E. siraeum* and *R. faecis* in females and *R. inulinivorans* in males and higher abundances of all 10 flagellated bacteria than the 500FG cohort, except *E. siraeum* (**Figure 3E**). By comparing the consistency of positive correlations between the FLA pathway and HDL-C in the females of the 500FG cohort and the males of the Chinese cohort, *E. rectale*, *L. pectinoschiza*, *R. intestinalis* and *R. inulinivorans* appeared to be critical for the positive correlation with HDL-C levels (**Figure 3F**). Taken together, the capacity of bacteria to produce flagellins was positively associated with HDL-C levels in humans, but not all flagellated bacteria provided the same degree of benefits, and the types and penetration of flagellins also play important roles.

### Detections of TLR5 but not NLRC4/NAIP in human liver

**Figure 4:**
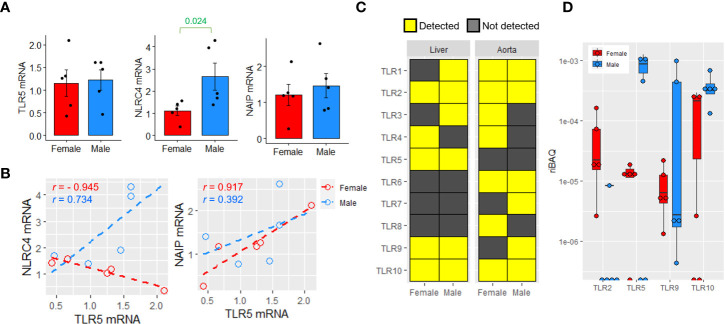
Sex difference in the expression of flagellin-engaging receptors. **(A)** Relative mRNA expression of *TLR5*, *NAIP* and *NLRC4* in female and male livers (*n*=5 females, *n*=6 males). Two-sided unpaired *t* test. **(B)** Correlation of the mRNA expressions of *NAIP* and *NLRC4* with *TLR5* in livers. Pearson correlation. **(C)** The detection of various TLRs in human livers and abdominal aortic lesions in females and males (*n*=5 per sex for livers, *n*=3 per sex for aortic lesions). **(D)** The relative intensity-based absolute quantification (riBAQ) of different TLRs in human liver samples (*n*=5 per sex).

Hepatic TLR5 responds to gut bacteria-derived flagellins in mice (Yiu et al., 2020). Therefore, the presence of flagellin-engaging receptors, including TLR5 and NAIP/NLRC4, was examined. Both *TLR5* and *NLRC4* mRNA were expressed in human liver samples, and *NLRC4* mRNA was significantly higher in male liver samples (**Figure 4A**). *TLR5* showed a strong negative correlation with *NLRC4* and a positive correlation with *NAIP* in female livers; however, these strong correlations were absent in male livers (**Figure 4B**). Proteomic analysis revealed that TLR2, TLR5, TLR9, and TLR10 proteins were detectable in both female and male liver samples, whereas TLR6, TLR7, and TLR8 were undetectable (**Figure 4C**). TLR5 were detected in 4 out of 5 and 3 out of 5 liver samples from females and males, respectively (**Figure 4D**). In comparison, the aortic samples were subjected to the same analysis, but TLR5 was undetectable in samples of both sexes (**Figure 4C**). Because the whole liver tissue was used for the analysis, hepatic immune cells could contribute to the proteomic readout. Nonetheless, instead of the full spectrum of the TLR family, only certain TLRs were detected, suggesting that the proteomic readouts reflected the protein abundance in the hepatocytes rather than in the less populated non-parenchymal cells. Unlike TLR5, neither NAIP nor NLRC4 was detectable at the protein level in these 10 liver samples (**Supplementary Figure 4**).

## Discussion

Commensal bacteria have evolved various mechanisms to evade host surveillance. Certain flagellated bacteria or flagellins can escape the host’s mucosal immunity and the host may eventually evolve mechanisms that benefit from such evasion. Indeed, several species of gut bacteria, including *E. rectale*, have been found to be co-diversified with humans, which means a shared evolutionary history results in a reinforcement of commensal relationships across generations (Suzuki et al., 2022). A recent study reported that a new subclass of flagellins, coined silent flagellins, which are largely produced by members of the Lachnospiraceae family, have a lower affinity for dimeric TLR5 than well-studied potent flagellins such as those from the pathogenic *Salmonella enterica* yet retain their efficacy to stimulate TLR5 at high concentrations (Clasen et al., 2023). *E. rectale*, *L. pectinoschiza*, *R*. intestinalis, and *R. inulinivorans* belonged to this family (**Figure 3F**). It is likely that such silent properties allow flagellins from commensal bacteria to bypass the intestinal mucosal checkpoint but trigger hepatic TLR5 once they accumulate in the liver.

Although we found a sex difference in the positive correlation between FLA pathways and HDL-C level in both the 500FG and Chinese cohorts, the result was completely opposite (**Figures 1B**, **4A**). Most of the flagellated bacteria observed in the 500FG cohort were more enriched in the Chinese cohorts for both sexes. A unique difference was the higher abundance of *R. faecis* in the Chinese females. The FLA pathways from *R. faecis* showed the most consistent positive correlation with HDL-C levels in males in the 500FG cohort (**Figure 3A**). It is possible that *R. faecis* may have a negative impact on females, resulting in lower correlations between FLA pathways and HDL-C levels in Chinese females. Many factors contribute to the sex difference in HDL-C levels, and our study did not conclude that this difference was due to the sex difference in gut microbiota. Instead, by comparing the opposite phenomena in these two cohorts, we deduced that flagellated microbiota may be a common factor favorably affecting HDL-C levels. Moreover, the absence of *R. intestinalis*-derived flagellins in the liver samples, but a consistent positive correlation between its FLA pathway and HDL-C level, implies that this flagellated species may exert beneficial effects through other organs or mechanisms, for example, as a facilitator for the infiltration of flagellins from other species.

It is generally believed that TLRs aggravate cardiovascular and metabolic disorders by inducing an inflammatory response. However, the effects of TLR5 are rather unconventional; for example, a genetic study reported that polymorphism of TLR5 (rs5744174) is negatively associated with HDL-C levels (Gu et al., 2016). Our previous study showed that recombinant flagellins could stimulate TLR5 to produce ApoA1 in mouse and human hepatocytes, suggesting a beneficial metabolic action in non-immune cells (Yiu et al., 2020). On the other hand, flagellin can activate NAIP/NLRC4 inflammasomes resulting production and secretion of proinflammatory cytokines (Vance, 2015). The detection of TLR5 but not NLRC4/NAIP in this study using human samples supports the notion of targeting hepatic TLR5 to increase HDL-C levels without the concern of the cross-activation of NAIP/NLRC4, and the candidates for flagellin-mimetics should be explored.

A limitation of this study is the different sources of human samples, although the gut microbiota data and liver samples originated from Europe. The lack of fecal samples for the examination of actual flagellin protein abundance prevented us from validating whether the undetected flagellins from certain species were due to the absence of bacteria or impermeability to the host. Future experimental studies in humans will reveal the mechanism by which gut microbiota-derived flagellins enter circulation.

In conclusion, through a multi-omics approach to investigate the sex differences in gut microbiota in two population-based cohorts, we identified that commensal flagellated microbiota in the gut could partially explain the host HDL-C level, which, along with the detection of their flagellins in liver tissues, confirms our previous *in vitro* observations in human primary hepatocytes that flagellins can stimulate ApoA1 production by engaging TLR5 (Yiu et al., 2020). We believe that this favorable interaction between the gut microbiota and the host will inspire the development of drugs and drug targets for increasing ApoA1 and HDL-C levels.

## Materials and methods

### Population cohorts

The 500 Functional Genomics (500FG) project was initiated in 2013 and 534 healthy adult volunteers were recruited from the Netherlands (Asnicar et al., 2021). Inclusion criteria were (1) older than 18 years of age and (2) Western European descent, and exclusion criteria were (1) pregnancy/breastfeeding, (2) chronic or acute disease at the time of assessment, and (3) use of chronic or acute medication during the last month before the study. Only samples with shotgun metagenomic data on the gut microbiome, complete physiological parameters, and lipid profile were included in our study, yielding 347 samples (*n*=193 females, *n*=154 males) in the final analyses. The Chinese cohort consisted of 942 healthy participants from six ethnicities in urban and rural China (Sun et al., 2021). After excluding participants (1) without HDL-C level measurement and shotgun metagenomic data on the gut microbiome, (2) who took antibiotics, Western medicine, or traditional Chinese medicine, and (3) living in rural areas, 316 samples (*n*=213 females, *n*=103 males) were analyzed in our study. Data from participants within the 90^th^ percentile based on the distributions of lipoprotein levels were used for the correlation analysis for both cohorts. The use of these public repositories for human data was approved by the Institutional Review Board of The University of Hong Kong/Hospital Authority Hong Kong West Cluster (UW 21-143).

### Human tissues

Human liver tissues were purchased from BioIVT (France) and were free of pathology and infection with HBV/HCV/HIV. Specimens of retroperitoneal adipose tissue and aortic walls were obtained from patients undergoing open aortic replacement at the Division of Vascular Surgery, University Medical Center, Johannes Gutenberg University (Mainz, Germany). All patients provided informed consent, and the institutional review board approval was waived (use of excess/discarded material). Specimens were snap-frozen in liquid nitrogen immediately after collection. Ethical approval for the use of these human tissues was granted by the institutional review board of The University of Hong Kong/Hospital Authority Hong Kong West Cluster (UW 22-075).

### Gut microbiome profiling

High-quality metagenomic data from the 500FG and Chinese cohorts were downloaded from the Sequence Read Archive of the NCBI. The sequence files were first preprocessed using KneadData (Huttenhower Lab, Harvard University, USA) with the default settings, including the removal of human contaminant sequences (hg37dec_v0.1) identified by bowtie2 using the very-sensitive mode, reads with low-quality reads (Q<20), and fragmented short reads (<50 bp). The taxonomic profile was determined using MetaPhlAn 3.0, (Huttenhower Lab, Harvard University, USA). This version was built using 99,237 reference genomes representing 16,797 species retrieved from GenBank, as of January 2019 (Truong et al., 2017). Functional profiling was performed using HUMAnN 3.0 (The Huttenhower Lab, Harvard University, USA) (Beghini et al., 2021). In brief, the cleaned reads were first mapped to clade-specific marker genes to identify community species using MetaPhlAn 3.0, followed by mapping to the pangenomes of the identified species (ChocoPhlAn database). The unmapped reads were aligned to a non-redundant database, Uniref90 (version 201901b). All aligned results were then estimated with the total gene family abundance per species and community based on alignment quality, gene length, and gene coverage. Functional enrichment analysis was performed by grouping the gene families using Gene Ontology (GO) annotations. The abundance in counts per million (cpm) was analyzed in this study.

### Proteomic identification using LC-MS/MS

Human liver and aortic lesion protein lysates were separated on SDS-PAGE gels and visualized with Coomassie stain. The positions at which flagellins were detected using anti-flagellin antibodies from Covalab Inc. (France) and Abcam Inc. (UK) (approximately between 26 to 43 kDa) were excised for flagellin identification, whereas proteins > 72 kDa were excised for identification of TLRs and NLRs. The gel slices were then subjected to in-gel digestion with trypsin, followed by LC-MS/MS analysis at the Center for PanorOmic Sciences, HKU. Raw mass spectrometry data were processed using MaxQuant 2.0.1.0 (Max Planck Institute for Biochemistry, Germany) (Tyanova et al., 2016).

(See **Supplemental Material** for additional details).

### Statistical analyses

Statistical analyses were performed using R software version 4.0.5. Differences in gut microbiota patterns were evaluated by permutational ANOVA (PERMANOVA) using the adonis function from the vegan package in R. Normality was checked using the Shapiro-Wilk test or Kolmogorov-Smirnov test. Equal variances were checked using Levene’s test. Means were compared using a two-sided Student’s t-test or Mann-Whitney U test for samples with or without normal distribution, respectively. Spearman partial correlation analysis was performed using ppcor packing in R, and the cutoff at the 90^th^ percentile was applied to exclude extreme values. *P* values were adjusted for multiple testing based on the Benjamini-Hochberg method and reported as *q* values. Significance was defined as *P*<0.05 and *q*<0.2 when multiple testing was applied.

## Data availability statement

The gut metagenome data are available at NCBI SRA BioProject under accession ID PRJNA319574 (500FG cohort) and PRJNA588513 (Chinese cohort). The lipid and cytokine profiles of the 500FG cohort are available at https://hfgp.bbmri.nl/while the data for the Chinese cohort are available in the original paper (Sun et al., 2021). Proteomics data were deposited in the Proteomics Identification Database under accession ID PXD041941. The remaining data supporting the findings of this study are available from the corresponding authors upon reasonable request. Source codes for KneadData, MetaPhlAn 3.0 and HUMAnN 3.0 are available at https://github.com/biobakery/ (Truong et al., 2017; Beghini et al., 2021).

## Ethics statement

The studies involving humans were approved by Institutional Review Board of The University of Hong Kong/Hospital Authority Hong Kong West Cluster (UW 21-143 and UW 22-075). The studies were conducted in accordance with the local legislation and institutional requirements. The human samples used in this study were acquired from a by- product of routine care or industry. Written informed consent for participation was not required from the participants or the participants’ legal guardians/next of kin in accordance with the national legislation and institutional requirements.

## Author contributions

JY: Conceptualization, Data curation, Formal Analysis, Investigation, Methodology, Validation, Writing – original draft, Writing – review & editing. JC: Investigation, Writing – review & editing. SC: Investigation, Writing – review & editing. KC: Investigation, Writing – review & editing. CC: Software, Writing – review & editing. EM: Investigation, Writing – review & editing. RS: Methodology, Writing – review & editing. BD: Resources, Writing – review & editing. CWW: Conceptualization, Data curation, Formal Analysis, Funding acquisition, Investigation, Methodology, Project administration, Resources, Supervision, Validation, Visualization, Writing – original draft, Writing – review & editing.

## References

[B1] Aleksander S. A. Balhoff J. Carbon S. Cherry J. M. Drabkin H. J. Ebert D.. (2023). The gene ontology knowledgebase in 2023. Genetics 224 (1): 1–14. doi: 10.1093/genetics/iyad031 PMC10158837 36866529

[B2] Ashburner M. Ball C. A. Blake J. A. Botstein D. Butler H. Cherry J. M.. (2000). Gene Ontology: tool for the unification of biology. Nat. Genet. 25 (1), 25–29. doi: 10.1038/75556 10802651 PMC3037419

[B3] Asnicar F. Berry S. E. Valdes A. M. Nguyen L. H. Piccinno G. Drew D. A.. (2021). Microbiome connections with host metabolism and habitual diet from 1,098 deeply phenotyped individuals. Nat. Med. 27 (2), 321–332. doi: 10.1038/s41591-020-01183-8 33432175 PMC8353542

[B4] Beghini F. McIver L. J. Blanco-Míguez A. Dubois L. Asnicar F. Maharjan S.. (2021). Integrating taxonomic, functional, and strain-level profiling of diverse microbial communities with biobakery 3. Elife 10, e65088. doi: 10.7554/eLife.65088 33944776 PMC8096432

[B5] Clasen S. J. Bell M. E. W. Borbón A. Lee D. H. Henseler Z. M. de la Cuesta-Zuluaga J.. (2023). Silent recognition of flagellins from human gut commensal bacteria by Toll-like receptor 5. Sci. Immunol. 8 (79): 1–14. doi: 10.1126/sciimmunol.abq7001 36608151

[B6] Ding T. Schloss P. D. (2014). Dynamics and associations of microbial community types across the human body. Nature 509 (7500), 357–360. doi: 10.1038/nature13178 24739969 PMC4139711

[B7] Fu J. Bonder M. J. Cenit M. C. Tigchelaar E. F. Maatman A. Dekens J. A.. (2015). The gut microbiome contributes to a substantial proportion of the variation in blood lipids. Circ. Res. 117 (9), 817–824. doi: 10.1161/CIRCRESAHA.115.306807 26358192 PMC4596485

[B8] Godsland I. F. Crook D. Simpson R. Proudler T. Felton C. Lees B.. (1990). The effects of different formulations of oral contraceptive agents on lipid and carbohydrate metabolism. New Engl. J. Med. 323 (20): 1375–1381. doi: 10.1056/nejm199011153232003 2146499

[B9] Gu L. Huang J. Tan J. Wei Q. Jiang H. Shen T.. (2016). Impact of TLR5 rs5744174 on stroke risk, gene expression and on inflammatory cytokines, and lipid levels in stroke patients. Neurol. Sci. 37 (9), 1537–1544. doi: 10.1007/s10072-016-2607-9 27262705

[B10] Koeth R. A. Wang Z. Levison B. S. Buffa J. A. Org E. Sheehy B. T.. (2013). Intestinal microbiota metabolism of L-carnitine, a nutrient in red meat, promotes atherosclerosis. Nat. Med. 19 (5), 576–585. doi: 10.1038/nm.3145 23563705 PMC3650111

[B11] Li J. Lin S. Vanhoutte P. M. Woo C. W. Xu A. (2016). Akkermansia muciniphila protects against atherosclerosis by preventing metabolic endotoxemia-induced inflammation in apoe-/- mice. Circulation 133 (24), 2434–2446. doi: 10.1161/CIRCULATIONAHA.115.019645 27143680

[B12] Ouimet M. Barrett T. J. Fisher E. A. (2019). HDL and reverse cholesterol transport. Circ. Res. 124 (10), 1505–1518. doi: 10.1161/CIRCRESAHA.119.312617 31071007 PMC6813799

[B13] Schirmer M. Smeekens S. P. Vlamakis H. Jaeger M. Oosting M. Franzosa E. A.. (2016). Linking the human gut microbiome to inflammatory cytokine production capacity. Cell 167 (4), 1125–1136 e8. doi: 10.1016/j.cell.2016.10.020 27814509 PMC5131922

[B14] Sinha T. Vich Vila A. Garmaeva S. Jankipersadsing S. A. Imhann F. Collij V.. (2019). Analysis of 1135 gut metagenomes identifies sex-specific resistome profiles. Gut Microbes 10 (3), 358–366. doi: 10.1080/19490976.2018.1528822 30373468 PMC6546312

[B15] Sun Y. Zuo T. Cheung C. P. Gu W. Wan Y. Zhang F.. (2021). Population-level configurations of gut mycobiome across 6 ethnicities in urban and rural China. Gastroenterology 160 (1), 272–286 e11. doi: 10.1053/j.gastro.2020.09.014 32956679

[B16] Suzuki T. A. Fitzstevens J. L. Schmidt V. T. Enav H. Huus K. E. Ngwese M. M.. (2022). Codiversification of gut microbiota with humans. Sci. (1979) 377 (6612): 1328–1332. doi: 10.1126/science.abm7759 PMC10777373 36108023

[B17] Truong D. T. Tett A. Pasolli E. Huttenhower C. Segata N. (2017). Microbial strain-level population structure & genetic diversity from metagenomes. Genome Res. 27 (4): 626–638. doi: 10.1101/gr.216242.116 28167665 PMC5378180

[B18] Tyanova S. Temu T. Cox J. (2016). The MaxQuant computational platform for mass spectrometry-based shotgun proteomics. Nat. Protoc. 11 (12): 2301–2319. doi: 10.1038/nprot.2016.136 27809316

[B19] Vance R. E. (2015). The NAIP/NLRC4 inflammasomes. Curr. Opin. Immunol. 32, 84–89. doi: 10.1016/j.coi.2015.01.010 25621709 PMC4336817

[B20] Wang Z. Klipfell E. Bennett B. J. Koeth R. Levison B. S. Dugar B.. (2011). Gut flora metabolism of phosphatidylcholine promotes cardiovascular disease. Nature 472 (7341), 57–63. doi: 10.1038/nature09922 21475195 PMC3086762

[B21] Yiu J. H. C. Chan K. S. Cheung J. Li J. Liu Y. Wang Y.. (2020). Gut microbiota-associated activation of TLR5 induces apolipoprotein A1 production in the liver. Circ. Res. 127 (10), 1236–1252. doi: 10.1161/CIRCRESAHA.120.317362 32820707 PMC7580858

