# Supplementary material for: The association between flagellin producers in the gut microbiota and HDL-C level in humans
Source: Front Microbiomes. 2023 Nov 9;2:1287369. doi: 10.3389/frmbi.2023.1287369 (PMC12993611; doi:10.3389/frmbi.2023.1287369)
Supplement: Supplementary file 1 [file DataSheet_1.pdf]

Supplementary material

The Association between Flagellin Producers in the Gut Microbiota and HDL-C Level in Humans

Jensen H. C. Yiu, Jieling Cai, Samson W. M. Cheung, Karie Tsz-Ching Chin, Chi Fai Chan, Edward S.C. Ma, Rakesh Sharma, Bernhard Dorweiler, Connie W. Woo*

Supplementary materials include:

- Supplementary methods
- Supplementary Figures 1 to 4

Supplemental Methods

Protein identification using LC-MS/MS proteomic analysis

The trypsin-digested gel slices were subjected to reduction and alkylation by 10 mM tris(2-carboxyethyl)phosphine (TCEP) and 55 mM 2-chloroacetamide (CAA), respectively following by incubation with trypsin (1 ng/µl) overnight at 37 °C. Subsequent tryptic peptides were extracted from the gel with 50% acetonitrile (ACN)/5% formic acid (FA) and 100% ACN sequentially and desalted. The eluted peptide mixture was loaded onto an Aurora C18 UHPLC column (75 µm i.d. × 25 cm length × 1.6 µm particle size) (IonOpticks, Australia) and separated using a linear gradient of 2-30% of buffer B (0.1% FA in ACN) at a flow rate of 300 nl/min buffer A (0.1% FA and 2% ACN in H_2O) for 100 min on nanoElute Nano-Flow UHPLC System coupled to timsTOF Pro mass spectrometer (Bruker, USA). MS data was collected over a *m*/*z* range of 100 to 1700, and MS/MS range of 100 to 1700. During MS/MS data collection, each TIMS cycle was 1.1 s and included 1 MS + an average of 10 PASEF MS/MS scans.

Sequence identification and validation

Detection of flagellins: The raw MS spectra were searched against human UniProt FASTA database (Apr 2020) containing 74,824 entries or a customized database containing 137 flagellin proteins identified in the gut microbiota analysis of the 500FG cohort belonging to the “Bacterial Flagellin Family” according to UniProt with duplicates removed, using MaxQuant (version 2.0.1.0) with the settings as below: oxidized methionine (M), acetylation (Protein N-term) were selected as dynamic modifications, and carbamidomethyl (C) as fixed modifications with minimum peptide length of 7 amino acids was enabled. Gut microbiota profile exhibits a great extent of inter-individual variation and the infiltrated flagellin proteins are, therefore, anticipated to be likely from different species and therefore distinct protein sequences across individuals. In MS spectral analysis, including multiple raw data files in a single run allows identification of common protein groups with a great accuracy and reliability; however, the

consequential drawback is the potential erroneous neglection of proteins present in few samples with relatively lower abundance owing to the stricter FDR control. As a result, the raw MS spectra were analyzed individually here to identify the unique flagellin profiles in each liver sample.

Validation of proteomic technique for flagellin identification: To test whether the LC-MS/MS technique could correctly identify the flagellin protein, we spiked in known amounts of flagellin from *Bacillus subtilis* subsp. spizizenii ATCC 6633 (Taxonomy ID: 703612; Protein accession: EFG93392) (InvivoGen, USA), a species that was not identified in the 500FG cohort, followed by proteomic analysis, in a liver sample. This flagellin was not identified in any sample during the gut microbiota nor in the proteomic analyses. In both the spiked samples, the *Bacillus subtilis*-flagellin was detected along with other flagellin species. The flagellin from *B. subtilis* was detected in the spiked samples in a concentration-dependent manner. The corresponding intensity-based absolute quantification (iBAQ) values were 4270.8 and 212110 when 1 ng and 50 ng *Bacillus subtilis*-flagellins were spiked, respectively, whereas the iBAQ value was 4176600 when 1500 ng *Bacillus subtilis*-flagellin alone was subjected to proteomics measurement alone (R^2 = 0.9998, linear regression). The linear concentration-dependent increase in detection demonstrated the effectiveness of such technique to detect flagellin proteins in our samples. In addition, to verify the identified flagellin peptides in human liver were not from the endogenous proteins, we aligned them to the human proteome (Taxonomy ID: 9606) using Protein BLAST (National Center for Biotechnology Information , USA). Among the 101 flagellin peptides identified in the 26-kDa samples and the 89 peptides in the 26–43-kDa samples, none of them could completely align to any human protein. Therefore, it is unlikely for the identified flagellin peptides were from endogenous proteins.

Detection of TLRs and NLRs: The raw MS spectra were searched against databases containing all members from TLRs or NLRs, respectively, using MaxQuant. Confident proteins were

identified using a target-decoy approach with a reversed database, strict false discovery rate of 1% at peptide and peptide spectrum matches (PSMs) level with minimum ≥1 unique peptide [17] Unlike flagellins, the sequences of TLRs and NLRs present in different liver samples were anticipated to be identical or, at least, highly similar in case of any mutation. Therefore, the raw MS spectral data files were not analyzed individually to identify common protein groups across samples.

Western immunoblotting

Immunoblotting was conducted as described previously [9]. The anti-flagellin antibodies were purchased from Covalab Inc. (Catalogue no. pab0595) and Abcam Inc. (Catalogue no. ab93713). The membranes were reblotted with anti-β-actin (Catalogue no. A1978, Sigma-Aldrich, USA) for detecting differences in protein loading.

Real-time PCR

Total RNA from tissues was isolated using RNeasy Mini Kit (Qiagen). RNA was reverse-transcribed into cDNA using ImProm-II™ Reverse Transcription System (Promega, WI, USA). Real-time PCR was conducted using the SYBR green PCR reagent (Roche) and StepOnePlus™ System (Applied Biosystems, CA, USA). Amplicons were verified using agarose gel electrophoresis and Sanger sequencing. The sequences of the primers are listed in Supplementary Table 9.

A

	Female – Biological process
Motility	bacterial-type flagellum assembly
	bacterial-type flagellum organization
	bacterial-type flagellum-dependent cell motility
	regulation of bacterial-type flagellum assembly
Nucleobase	purine nucleobase metabolic process
	purine ribonucleotide biosynthetic process
	pyrimidine nucleobase metabolic process
	pyrimidine nucleoside metabolic process
	pyrimidine nucleotide metabolic process
	pyrimidine-containing compound salvage
	nicotinamide nucleotide metabolic process
	ATP biosynthetic process
Translation	arginyl-tRNA aminoacylation
	tRNA modification
	protein maturation
	peptide metabolic process
	negative regulation of translational initiation
Survival	plasmid maintenance
	response to redox state
	hydrogen peroxide catabolic process
	enterobacterial common antigen biosynthetic process
	cytokinin biosynthetic process
	polysaccharide metabolic process
CHO	'de novo' GDP-L-fucose biosynthetic process
	L-fucose catabolic process
	galactitol metabolic process
	rhamnose catabolic process
PL	phospholipid metabolic process
	galactosylceramide catabolic process
PO4	organic phosphonate catabolic process
	phosphorylation
Misc	glucuronate catabolic process
	glutathione catabolic process
	thiamine transport
	nor-spermidine biosynthetic process
	cytolysis by virus of host cell

Male – Biological process	
defense response to bacterium	Survival
defense response to virus	
double-strand break repair via homologous recombination	
maintenance of CRISPR repeat elements	
plasmid maintenance	
folic acid biosynthetic process	
organomercury catabolic process	
response to metal ion	
D-xylose metabolic process	CHO
galactarate catabolic process	
galactose catabolic process via UDP-galactose	
GDP-mannose biosynthetic process	
rhamnose catabolic process	
xylan catabolic process	
cellular biogenic amine metabolic process	Amine
allantoin catabolic process	
urea catabolic process	
proline transport	AA
prosthetic group biosynthetic process	
selenocysteine incorporation	
glyoxylate metabolic process	
nucleoside triphosphate catabolic process	Nu
IMP biosynthetic process	
protein kinase C-activating GPCR signaling pathway	Misc
glycerol metabolic process	

Female – Cellular Component
bacterial-type flagellum
bacterial-type flagellum basal body
bacterial-type flagellum basal body, MS ring
bacterial-type flagellum basal body, rod
bacterial-type flagellum filament
bacterial-type flagellum filament cap
bacterial-type flagellum hook

Female – Cellular Component
bicellular tight junction
glycine cleavage complex
glycine reductase complex
microtubule cytoskeleton
photosystem I

	Biological process	Cellular component	Molecular function
Number of GO terms positively correlated with HDL-C in females	36	11	53
Related to flagellum	4	7	1
Number GO terms negatively correlated with TG in males	25	5	70

B

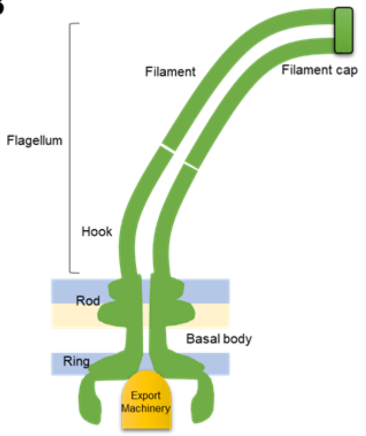

Supplementary Figure 1. Top 100 GO terms associated with HDL-C and TG levels in the 500FG cohort.

(A) Top 100 gene ontology (GO) terms that were positively correlated with HDL-C level in females (green) or top 100 terms that were negatively correlated with TG level in males (blue) in the 500FG cohort. GO terms have 3 sub-ontologies: biological process (BP), cellular component (CC) or molecular function (MF). BP terms were grouped according to their functions. Spearman correlation adjusted with age and BMI for all, and the use of oral contraceptives for females. AA, amino acids; CHO, carbohydrates; Misc, miscellaneous; Nu, nucleic acids; PL, phospholipids; PO4, phosphates. (B) Schematic diagram depicting the general structure of a bacterial flagellum.

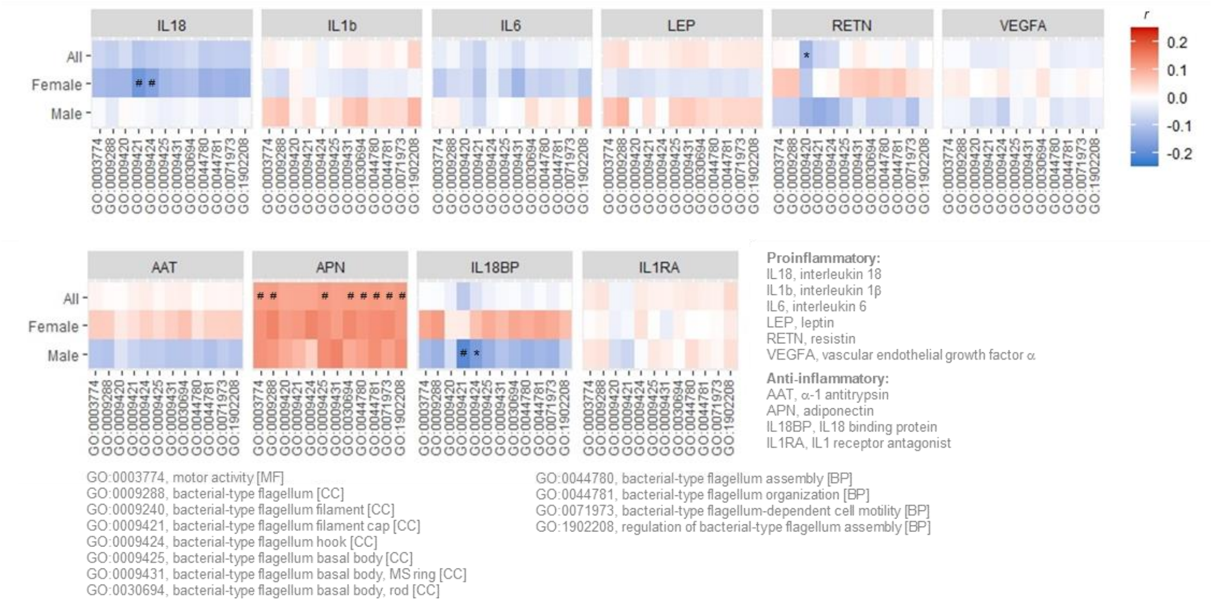

Supplementary Figure 2. Association between flagellum-related pathways and the anti-inflammatory profile in female participants of the 500FG cohort.

Correlations of the flagellum-related pathways with proinflammatory and anti-inflammatory molecules in participants of the 500FG cohort. Spearman correlation adjusted with age and BMI for all, and also the use of oral contraceptives for females. $^{*}P$<0.05, $^{\#}P$<0.05 and q<0.2. BP, biological process; CC, cellular component; MF, molecular function.

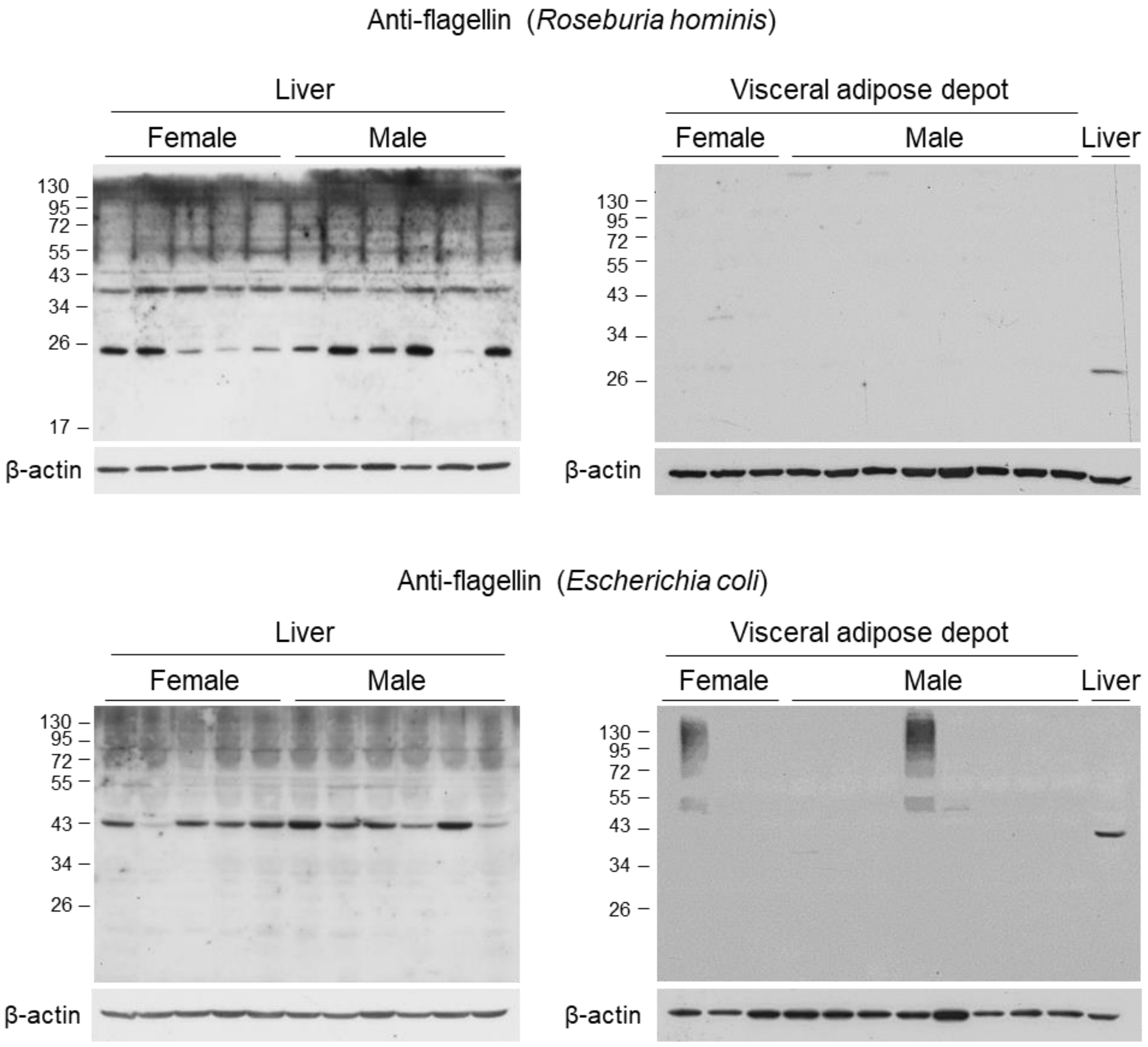

Supplementary Figure 3. Detection of flagellins in human liver samples.

Immunoblots of flagellins in human liver and visceral retroperitoneal adipose samples using two different anti-flagellin antibodies that were raised against *Roseburia hominis* from Firmicutes and *Escherichia coli* from Pseudomonadota, respectively. β-actin was used as a protein loading control. (*n*=5 females, *n*=6 males for liver, *n*=3 females, *n*=8 males for visceral adipose depots)

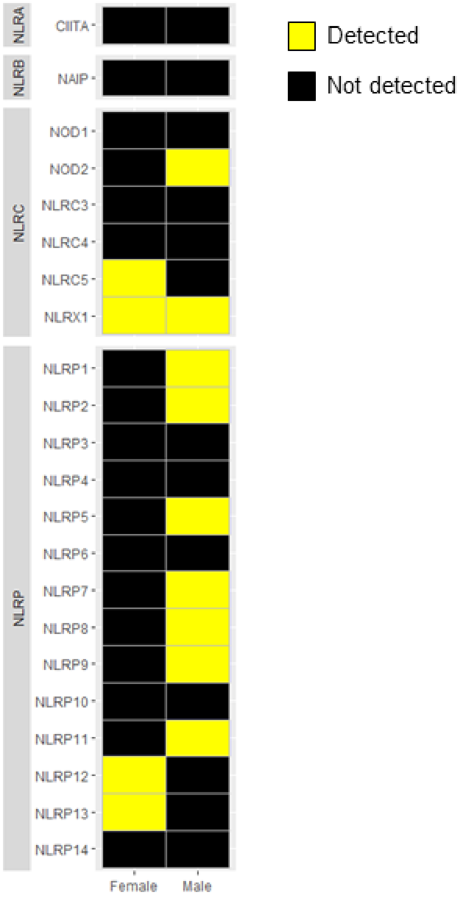

Supplementary Figure 4. Absence of NAIP and NLRC4 in human liver samples.

Expression profile of NOD-like receptors (NLRs) determined with LC-MS/MS proteomic analysis in human liver samples. (*n*=5 per sex)
